# Evaluating the Process of Patient Engagement: Insights from a Mixed‐Methods Evaluation of a Cancer Center Patient Advisory Group

**DOI:** 10.1111/hex.70581

**Published:** 2026-01-24

**Authors:** Lauren Kearney, Tracy Battaglia, Karina Verma, Sara Shusterman, Michelle Hall, Gemmae Fix, Katrina Steiling

**Affiliations:** ^1^ Boston University Chobanian & Avedisian School of Medicine Boston Massachusetts USA; ^2^ Boston Medical Center Boston Massachusetts USA; ^3^ Center for Health Optimization and Implementation Research (CHOIR), VA Boston Healthcare System Boston Massachusetts USA; ^4^ Yale Cancer Center, Yale University School of Medicine New Haven Connecticut USA; ^5^ John's Hopkins University School of Medicine Baltimore Maryland USA; ^6^ Center for Health Optimization and Implementation Research (CHOIR), VA Bedford Healthcare System Bedford Massachusetts USA

**Keywords:** mixed‐methods, oncology, patient engagement, patient navigation

## Abstract

**Background:**

Patient engagement is associated with improved care quality, better health outcomes, increased trust and satisfaction, and reduced costs. Patient engagement is recommended in cancer care. Patient advisory groups (PAGs) are a commonly used approach for engaging patients. However, more evidence is needed to understand how effectively PAGs support meaningful engagement, what factors shape that engagement, and how their contributions align with program goals. This study used a mixed‐methods approach to evaluate patient engagement within a PAG supporting an oncology quality improvement initiative.

**Methods:**

The Oncology Equity Alliance (OEA), a quality improvement initiative to improve care coordination and reduce time to treatment, established a PAG to engage patients throughout the initiative. We conducted a mixed‐method evaluation of PAG engagement. Focus groups with PAG members and qualitative interviews with OEA team members were rapidly analyzed to identify engagement successes and challenges, and to determine engagement principles to focus on for survey evaluation. Perceptions of PAG engagement were assessed via surveys of PAG members and OEA team members, using selected items from the Research Engagement Survey Tool (REST). Surveys were analyzed descriptively and according to the REST scoring scheme.

**Results:**

Focus groups (*n* = 2) and interviews (*n* = 3) identified key facilitators that supported engagement including deliberate coordination, mutual respect, a sense of belonging, and co‐learning. Engagement was also positively impacted by members' motivations for joining the PAG and practical and logistical considerations. Challenges included PAG members' desire for greater understanding of the project's impact, more agenda setting, and ongoing education about OEA core components. This qualitative data informed the selection of engagement principles of focus for quantitative evaluation using REST. A total of 80% of PAG members (*n* = 10) felt very engaged, with the degree of engagement corresponding to cooperation and collaboration domains; however, for individual survey items, an average of 20% of responses were marked by PAG members as “not applicable.”

**Conclusion:**

This mixed‐method evaluation found strong alignment between program goals and PAG member engagement, highlighted effective strategies, and identified addressable challenges. As patient engagement becomes more common in cancer care, applying these lessons is essential to advancing meaningful, person‐centered programs.

**Patient Contribution:**

This paper presents an evaluation of a PAG. In addition to being the study participants, the members of the patient advisory group participated in member checking to validate the study findings, ensuring that the interpretations reflected their experiences and perspectives.

## 1 Introduction

The growing burden of cancer, compounded by persistent challenges in access and gaps in care, underscores the need for patient‐centered initiatives that prioritize effective, holistic, and compassionate care [1, 2, 3]. Patient engagement plays a critical role in advancing these goals by uncovering blind spots, leveraging lived experience, and ensuring that programs and policies reflect the perspectives of those most affected. This inclusive approach has been shown to improve health outcomes, enhance care quality, increase patient satisfaction, and reduce healthcare costs [4, 5, 6, 7]. Patient engagement is also recommended by the United States National Cancer Institute and Commission on Cancer [8, 9]. One mechanism to facilitate this engagement is through patient advisory groups (PAGs), which provide structured opportunities for patients to contribute to the design, implementation, and evaluation of care initiatives [10].

While patient engagement through PAGs hold promise for advancing cancer care initiatives, there remains a lack of robust evidence that specifies best practices to engage participants in a meaningful and sustainable way [11, 12]. Existing research indicates that the level of involvement and engagement within PAGs can vary widely, which likely affects their overall effectiveness and ability to achieve intended outcomes [13, 14]. Therefore, formal evaluation of PAGs not only serves to improve engagement within a specific group but also generates insights that can strengthen the design and implementation of future PAGs.

Boston Medical Center Oncology Equity Alliance (OEA) is a quality improvement initiative to address challenges to implementation of evidence‐based patient navigation to improve oncology care coordination and time to treatment. This comprehensive, multi‐level initiative emphasizes partner engagement and accordingly, established a PAG to ensure the patient perspective is represented throughout planning, execution, and evaluation. Here, we present a mixed‐method study aimed at understanding the structure and operations of the OEA PAG while assessing areas of successful engagement and opportunities for future improvement.

## 2 Methods

### 2.1 The OEA

The OEA is a quality improvement initiative within the Boston Medical Center Cancer Center to improve patient navigation, a model of care that provides individualized, barrier‐focused support to guide them through the complexities of diagnosis, treatment, and follow‐up [15]. The comprehensive initiative emphasizes universal access to navigation, coordination across departments, the establishment and application of navigation policies, and active engagement of all care team members. The deliverable of the initiative is to establish a fully coordinated, efficient, and patient‐centered oncology navigation system that ensures timely connection to services, seamless communication across care teams, and consistent implementation of evidence‐based navigation practices.

### 2.2 The OEA PAG

One important goal of the OEA was to partner with patients throughout the planning, execution and evaluation of the quality improvement initiative. To facilitate this, 12 current Boston Medical Center patients who have had experience with cancer treatment at Boston Medical Center were recruited to participate in a PAG. The manager of cancer support programs (e.g., cancer support groups) distributed flyers and generated a list of potential participants who were contacted by an OEA team member to explain the objectives and logistics of PAG participation. Recruitment was purposive with attention to diversity regarding gender, race, and multilanguage ability. The 12 of 14 people approached agreed to participate in the PAG.

The PAG has met quarterly since January, 2023. Meetings are virtual, lasting 60–90 min. At the time of this study, seven PAG meetings over 2 years have occurred. A total of 10 of the original 12 members have continued as PAG members during this time. One member died, and one member stepped away after 1 year due to other commitments. PAG meetings focused on building rapport and included setting expectations, sharing member stories, and gathering input on specific components of the OEA (e.g., content for patient information cards about navigators, the organization and content of packets for newly diagnosed cancer patients, feedback on patient surveys for program evaluation). An OEA team member outreached to PAG members via phone call and email 1–2 times between each meeting. In addition, PAG members were sent holiday cards annually.

### 2.3 Participants

Our evaluation focused on the individuals involved with the OEA PAG, including the 10 current members of the PAG and the three key team members engaged in PAG activities.

#### 2.3.1 PAG Members

We recruited current members of the PAG after a warm hand‐off from the OEA team via email. Participants were informed of the study goals and purposes, including that this evaluation was separate from their role in the OEA PAG and was voluntary. Interviewer credentials were reviewed, and verbal consent was obtained prior to participation.

#### 2.3.2 OEA Team Members

Key team members were identified as people who have been highly engaged with PAG activities, including regular communication with PAG members, facilitation of PAG meetings, and support for logistical tasks such as managing payments and other administrative responsibilities. Participants were emailed to inform them of study goals and purposes and interviewer credentials. Verbal consent was obtained prior to participation.

### 2.4 Evaluation of the PAG

For this formative evaluation, we employed a mixed‐methods approach with qualitative (focus groups and interviews) and quantitative (surveys) data to identify determinants of engagement (qualitative) and perceived engagement in the PAG (quantitative). Focus groups were chosen to evaluate PAG members' experiences because they encourage discussion of shared perspectives and collective insights. Semi‐structured individual interviews with OEA team members were used to explore organizational processes and decision‐making in depth [16].

The study team was led by a pulmonologist and health services researcher (LEK); team members also included male and female pulmonologists, oncologists, and primary care health services researchers with qualitative research and community engagement experience. Between May and August 2024, focus groups, qualitative interviews, and surveys were completed by PAG members and key OEA team members engaged with the PAG. This study was approved by the Boston University and Boston Medical Center Institutional Review Boards and followed COREQ criteria.

#### 2.4.1 Qualitative Data

##### 2.4.1.1 Data Collection

Lead team member (LEK) conducted two focus groups with PAG members and three semi‐structured interviews with key OEA team members via videoconferencing. Focus groups lasted approximately 60 min and interviews lasted approximately 30 min. The interviewer had no prior relationship with participants. The focus group guide (Supporting Information: Appendix 1a) explored participants' experiences with the PAG including what worked well, barriers encountered, and suggestions for the future of this PAG and similar groups. The interview guide (Supporting Information: Appendix 1b) focused on team members role with the PAG, and perceived facilitators and barriers to PAG success. Focus groups and interviews were audio‐recorded and professionally transcribed. PAG members received a $50 gift card for participating in the focus group.

##### 2.4.1.2 Data Analysis

Focus groups and interviews were analyzed using a rapid content analysis approach to allow for rapid feedback to the OEA team about PAG engagement [17, 18]. The analysis team included four study team members with qualitative experience (LK, KV, SS, and MH). Focus groups and interview codebooks were developed based on the interview guides. For each participant group, analysis began by reviewing all transcripts, annotating for facilitators and challenges of engagement, and novel findings. Following individual review, the analysis team identified themes until consensus on the codebook was reached, one for each participant group. The codebook was then applied to each transcript followed by consensus meetings to discuss and resolve discrepancies until consensus was reached. Analysis of all transcripts was conducted in Microsoft Excel.

#### 2.4.2 Quantitative Data

##### 2.4.2.1 Data Collection

All PAG members and OEA team members were invited by email to complete the survey on RedCAP to evaluate their experience, regardless of participation in qualitative focus groups or interviews. All survey responses were collected anonymously. We utilized portions of the Research Engagement Survey Tool (REST) [19] to evaluate the level of perceived engagement in the PAG. We focused on how well the engagement principles were executed (quality of engagement as measured by the REST). Based on rapid analysis of the focus groups, four engagement principles were selected from the REST. This approach of focusing on four principles and their associated questions from the REST has been previously described [20]. The four engagement principles (EPs) selected were: EP1: Focus on community perspectives and determinants of health (four questions), EP4: Foster co‐learning, capacity building (four questions), and co‐benefit for all partners, EP6: Facilitate collaborative, equitable partnerships (four questions) and EP7: Involve all partners in the dissemination process (three questions). All REST survey questions use a 5‐point Likert scale, with higher ratings indicating greater quality. For all measures, not applicable was also an answer choice. The PAG member survey also included questions adapted from Hoke et al. [20] focusing on their experience with the PAG. PAG members received a $25 gift card for participating in the survey.

##### 2.4.2.2 Data Analysis

R version 4.1.0 was used for all analyses. For each group (PAG members and OEA team members), Likert‐scale responses were averaged at both the item level and within each REST engagement principle (EP1, EP4, EP6, and EP7). Results are reported as means and standard deviations, and group averages were compared descriptively.

We also sought to align the PAG participant responses with the levels of engagement as outlined by the REST scoring scheme for each item. The REST scoring scheme assigns each Likert scale answer for each survey item to one of five engagement levels: *Outreach & Education (least engagement), Consultation, Cooperation, Collaboration*, or *Partnership (most engagement)*. To determine engagement levels, each participant's response to each survey item was first categorized according to engagement level based on the REST scoring scheme rubric. Each participant survey then received one point for each response at each of the five engagement levels. For each participant, these points were summed for each of the five engagement levels (0–15) and converted into percentages (out of 15 points). Finally, across all participants, we calculated the average percentage and 95% confidence interval for each engagement level.

### 2.5 Member Checking

Member checking is a qualitative research technique in which researchers share data, findings, or interpretations with participants to verify accuracy, ensure credibility, and confirm that the analysis reflects participants' perspectives. [21] Prior to the completion of data analysis, preliminary results were shared with the PAG during a quarterly meeting. A slide presentation was used to present qualitative themes alongside corresponding quotes and selected EPs. The presentation was structured [21] so that each theme was introduced, followed by an opportunity for PAG members to give an overall “thumbs up” or “thumbs down” to the interpretations, and then engage in a guided discussion to provide clarification and feedback. Their insights were used to refine interpretations and contextualize survey findings as part of a broader evaluation approach, ensuring that results accurately reflected PAG member experiences.

## 3 Results

### 3.1 Qualitative Findings

We held two focus groups with PAG members (4 people participated in each focus group with eight participants total) and three qualitative interviews with OEA team members (*n* = 3). All quotes from focus groups are presented as (PAG Member‐Focus Group Number) and all quotes from qualitative interviews are presented as (OEA Team Member‐ Interview Number).

From the qualitative data, we identified several **key facilitators** that supported engagement. PAG members reported that they enjoyed being a part of the PAG, noting that PAGs are a good idea and should be replicated in other areas of care: *“I think it's a really, really, really, really, really good idea. Legitimizing this would be really good”* (PAG Member‐1).

Both PAG members and OEA team members highlighted **deliberate coordination**, which was evident in clearly defined team roles during meetings, structured facilitation, and check‐ins between meetings. As one team member explained, “[*During meetings], we have split up the roles… someone's looking at the chat, someone's facilitating through the slides and screen sharing… [and] I have my phone available so that I can help different people get it on*” (OEA Team Member‐2) A designated liaison provided personalized follow‐up through phone calls, emails, and reminders, which helped build trust and maintain momentum. As she described, “*Open communication and constant contact is really important”* (OEA Team Member‐3). Her colleagues echoed this, highlighting:

“she sends out holiday cards just really making sure that they don't forget. They only meet quarterly. So just helping people remember…That's really helped them be engaged”

(OEA Team Member‐2)

The frequency and quality of interactions contributed to a sense of **mutual respect** between PAG members and the OEA team. The OEA team members demonstrated a clear respect for the input from the PAG, with one saying, *“relying on the voice of those who are closest to the experience is extremely important and is sort of like the compass to a lot of the work”* (OEA Team Member‐2). Respect was noted as a main component of creating an environment for participation. This also contributed to a **sense of belonging** which was identified as an essential facilitator of meaningful participation. PAG members noted that *“people just have to feel comfortable”* and *“there's a sense of just belonging and acceptance, kind of like the support groups. The advisory group is equally accommodating through everyone's challenges”* (PAG Member‐2). This was also noted by OEA team members: *“what's surprising is how vulnerable they get in the meetings, and how open they are with their stories and the things that have impacted them in their lives with these diagnoses. And I think that sense of vulnerability really does show where the changes can be made and where real impacts can happen in the workflows and in getting people certain resources”* (OEA Team Member‐3).

PAG members had expected to contribute their own ideas to the OEA project but were surprised by the amount of information sharing and **co‐learning** that occurred. This was noted as a particularly unique aspect of participation. They also emphasized the value of learning from each other's perspectives and experiences, particularly noting how the diversity of cancer types and stages among members enriched the discussion. One person said, “*it's been good to share different views, point of views and everything with people and see what people have been through and what they think would be best for others coming through”* (PAG Member‐1).

**Motivations for joining the PAG** remained strong throughout participation. Many cited a desire to help other patients facing a cancer diagnosis as their primary reason for involvement. One member explained, “*It's good to be able to give information to help other people navigate their way through*” (PAG Member‐1). Some members also expressed a deep commitment to improving care at Boston Medical Center, inspired by the excellent treatment they received: *“They're the ones that shaped me—they were such good people. Unbelievable”* (PA GMember‐1). For others, the novelty and curiosity about the PAG itself motivated continued engagement: *“That was my motivation—I'm curious, and if you've got questions, ask. No question is dumb… You never know. So that kept me going*” (PAG Member‐2).

We also identified several **practical and logistical considerations** which influenced engagement. Participants appreciated the recruitment approach, which leveraged existing cancer support group lists and ensured that members were already comfortable sharing their experiences. As one OEA team member noted, “*Something that really helped us in the beginning was, the way that we recruited these folk are through the cancer survivor groups. So they were already in a sense committed to engaging and talking about their stories in certain spaces, so moving that into a different space wasn't too much of a leap for them*” (OEA Team Member‐1). However, team members also expressed concerns that this approach might have introduced selection bias, potentially excluding important perspectives. One observed, “*All patients were referred by the support group coordinator, so their willingness to participate may reflect a more positive experience. We might be missing the voices of those who had negative experiences or who don't engage with support groups*” (OEA Team Member‐3).

PAG members had mixed feedback on meeting logistics such as group size, frequency of meetings, and virtual versus in‐person participation. Small groups were preferred for deeper sharing *(“a small group is better than a whole big group. Because then you learn more, you hear more and to hear from everybody” PAG Member‐2)*, while larger groups were valued for representing a breadth of experiences (“*I'd like a little bit more people involved because I just think that one more diversity, more like everyone has different types of cancers and there's just a lot of input needed”*) (PAG Member‐2). Quarterly meetings fit busy schedules but made it harder to track progress over time: “*I'd like to see it more often. Waiting every three months to do something… now I forget what I have for breakfast some days. I keep going back to Chemo brain*” (PAG Member‐1). Finally, hybrid options for meeting were felt to be preferable to accommodate both those who wanted to meet in person and participate virtually: *“this is my opinion for those that want to meet me, then they can have it virtual too for the people that can't make it. So they should have it both”* (PAG Member‐2).

Although both PAG members and OEA team members noted several strong facilitators of engagement and most participants remained involved throughout the 2 years, three key themes emerged related to **challenges** in sustaining engagement. PAG members relayed that while they could recall the very specific programmatic feedback that they had provided, they were left wondering about the broader impact or **big picture** of their participation. One member noted: “*My interest is really in seeing what the endgame is going to be*” (PAG Member‐2). They recommended sharing updates on both how their input was influencing the broader project and how the project was progressing to bridge this gap: “*If there is a big picture like that and there ‐‐ if there are spoils to be had we want to join with the Pirates*” (PAG Member‐1).

One barrier to engagement that emerged was a lack of clarity and structure around **agenda setting**. While OEA team members noted an effort to set an agenda, stating, “for the actual meetings, in order to really get what we're looking for from the group, we really try and have a good, structured agenda for everyone (OEA Team Member‐1), some PAG members expressed a desire for greater transparency and preparation ahead of meetings, including the opportunity to review and reflect on discussion topics in advance.” Another noted some confusion about their role, stating, “*Well, I think in some ways there might be some questions about what you guys really want from us…*” (PAG Member‐1) and pointed to how discussions sometimes veered away from their intended focus. In response to these concerns, the OEA team adapted by sharing materials and proposed agendas in advance of meetings.

Finally, participants highlighted a need for **ongoing education** about the OEA program, particularly the role of patient navigators. Several PAG members expressed confusion about how navigators were integrated into care, what their specific responsibilities were, and if there was a consistent point of contact for patients. These reflections underscored the need for reinforcement of the navigator's role, given their central function within the OEA program that PAG members were actively helping to shape.

### 3.2 Quantitative Data

All 10 engaged PAG members completed the survey and 2/3 OEA team members completed the survey.

#### 3.2.1 PAG Engagement Survey

Eighty percent (8/10) of PAG members reported feeling *very engaged* in the advisory group, while the remaining 20% reported *moderate engagement*. Ratings of specific dimensions of engagement were consistently high, with average scores ranging from 3.2 to 3.6 on a 4‐point scale (1 = strongly disagree, 4 = strongly agree). Respondents strongly agreed that they could contribute productively (mean = 3.6, range = (3–4)), felt valued as team members (mean = 3.6, range = (3–4)), and that meetings were worth their time (mean = 3.4, range = (3–4)). Slightly lower but still favorable scores were observed for perceptions of being able to use their expertise (mean = 3.2, range = (2–4)) and appropriate compensation (mean = 3.3, range = (3–4)) (Supporting Information: Table S1).

#### 3.2.2 Overall Quality of Engagement‐ REST

**Figure 1:**
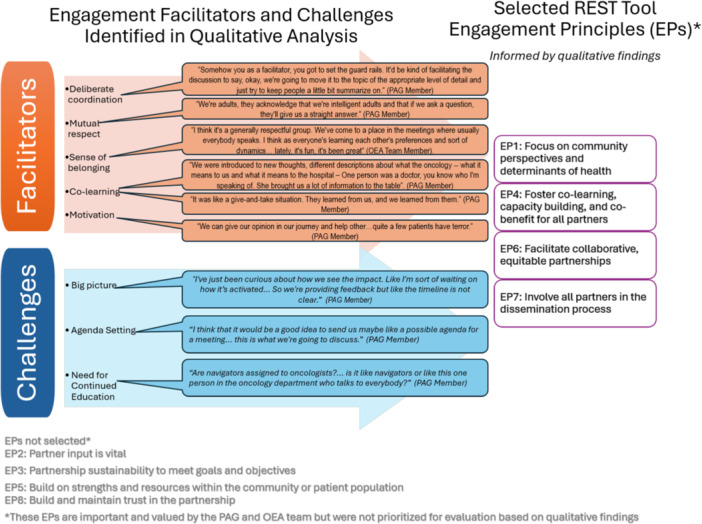
Mixed Method Evaluation of Patient Advisory Group Engagement. EPs not selected*. EP2: Partner input is vital. EP3: Partnership sustainability to meet goals and objectives. EP5: Build on strengths and resources within the community or patient population EP8: Build and maintain trust in the partnership. *These EPs are important and valued by the PAG and OEA team but were not prioritized for evaluation based on qualitative findings.

Using REST, PAG members (*n* = 10) and OEA team members (*n* = 2) evaluated overall quality of engagement (how well). The qualitative findings provided critical insights that guided the selection of the REST areas of focus for this evaluation (Figure 1), ensuring that the evaluation priorities were grounded in participants' experiences, perspectives, and identified needs. During focus groups, participants emphasized the importance of grounding the work in community perspectives (EP1), the reciprocal nature of the group's discussions reflecting a strong culture of co‐learning (EP4), the presence of collaborative and equitable partnerships (EP6), and the desire to involve all partners in dissemination (EP7).

**Figure 2:**
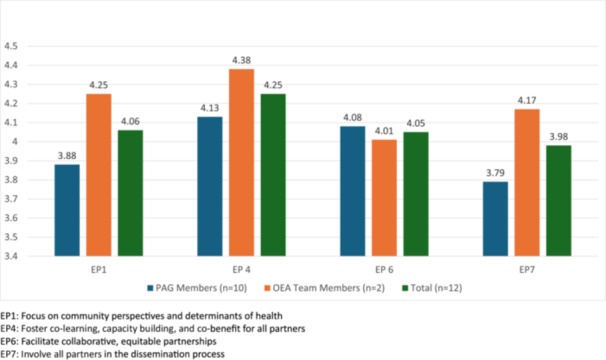
Results from REST survey quality of engagement for each engagement principle (reported as means). EP1: Focus on community perspectives and determinants of health. EP4: Foster co‐learning, capacity building, and co‐benefit for all partners. EP6: Facilitate collaborative, equitable partnerships. EP7: Involve all partners in the dissemination process.

PAG members and OEA team members responded similarly across EPs though OEA team members rated engagement quality higher than PAG members across all four EPs. The difference in groups for EP 4 (Foster co‐learning, capacity building and co‐benefit for all partners) was 0.25. The two groups responded most similarly in their experience of EP 6 (Facilitate collaborative, equitable partnerships), difference of 0.07, and most divergently about EP 7 (Involve all partners in the dissemination process), difference of 0.38, and EP 1 (Focus on community perspectives and determinants of health), difference of 0.37 (Figure 2).

Using the REST scoring scheme to evaluate the PAG members surveys, we found that engagement primarily occurred at the levels of collaboration (mean = 40% of responses) and cooperation (mean = 24%). For all participants, the highest proportion of responses were also connected to collaboration or cooperation (Table 1). Of note, all participants answered N/A to a portion of questions (mean = 20%, range = 6.7%–60%).

**Table 1:** Results of REST survey quality of engagement reported per REST scoring scheme.

Participant	N/A %	Outreach & education %	Consultation %	Cooperation %	Collaboration %	Partnership %
1	60.0	0	6.7	13.3	**20.0**	0
2	6.7	13.3	0	20.0	**60.0**	0
3	40.0	6.7	0	6.7	**40.0**	6.7
4	20.0	20.0	6. 7	20.0	**33.3**	0
5	6.7	13.3	13.3	**66.7**	0	0
6	20.0	6.7	0	6.7	**60.0**	6.7
7	6.7	13.3	6. 7	**73.3**	0	0
8	13.3	6.7	0	13.3	**66.7**	0
9	13.3	6.7	6. 7	6.7	**66.7**	0
10	13.3	6.7	0	13.3	**53.3**	13.3
Average (95% CI)	20 (7.7, 32.3)	9.3 (5.8–12.8)	4.0 (1.1–6.9)	24 (8.6–39.4)	**40 (23.9–56.1)**	2.7 (0–5.4)

*Note:* Using the REST rubric, each Likert‐scale response was mapped to one of five engagement levels ranging from Outreach & Education to Partnership. For each participant, responses were scored and summed across engagement levels, converted to percentages, and then averaged across participants to calculate the mean percentage and 95% confidence interval for each level.

All values are a percentage of total responses (15) that met each of the levels of engagement as described by the REST scoring guide. Bold indicates the highest percentage for each individual and average across the group.

## 4 Discussion

In this evaluation of a PAG, we explored facilitators and challenges of member engagement to identify key drivers of meaningful participation and to illustrate the value of a mixed‐method approach for evaluating engagement in PAGs. Our findings show that engagement was affected by both relational and structural elements. Perceived engagement among PAG members aligned most closely with the domains of cooperation and collaboration, suggesting that OEA PAG model supported high levels of engagement that aligned with the overarching goals of the OEA initiative. At the same time, we identified ongoing, but addressable, challenges including maintaining a focus on the big picture, setting shared agendas, and providing continuous education on essential program components. These findings underscore the importance of evaluation and feedback to support the continuous improvement of PAG operations and their impact.

Patient engagement is valued for its potential to improve care quality, health outcomes, satisfaction, trust, and even reduce healthcare costs [4, 5, 6, 7]. However, the science of engagement is still evolving, and much of what is promoted as best practice is based more on experience than evidence [22]. The OEA PAG demonstrated sustained engagement, with 10 out of 12 members maintaining involvement over the 2‐years from initiation to this study. This is notable given the common challenges of long‐term participation, which can be exacerbated when members have complex medical illness such as cancer [23, 24]. The REST responses reflected this sustained participation, with most falling in the cooperation and collaboration domains. Taken together, these findings suggest that practices employed in the OEA PAG such as deliberate facilitation (both during and between meetings), fostering mutual respect and belonging, and prioritizing co‐learning may be central to effective engagement. Additionally, thoughtful attention to recruitment and meeting logistics appears critical to sustaining participation. As evidence continues to emerge, these insights contribute to the evolving understanding of best practices for engaging patients in long‐term, impactful ways.

A principle of patient‐engagement is ensuring that the level of patient engagement aligns with the project's goals [25, 26]. As the OEA PAG aims to partner with patients throughout planning, execution, and evaluation of the quality improvement initiative, emphasis on cooperation (i.e., researchers seeking help, not just advice) and collaboration (i.e., involving patients in all project aspects) supports this goal. Our findings underscore the critical role of engagement assessment tools in ensuring that patients' perceived engagement meaningfully aligns with project goals [19]. Furthermore, an in‐depth assessment can reveal blind spots for those leading a PAG, as demonstrated by the discordance between PAG members' and OEA team members' impressions regarding agenda setting, an issue that has been specifically addressed since this evaluation.

This study underscores the value of a mixed‐methods approach to understanding patient engagement where qualitative insights provide depth and context, and quantitative tools reveal patterns, allowing the two methods to complement and inform each other. While prior research has established the importance of trust and supportive structures for meaningful patient engagement [27, 28, 29], our qualitative findings deepen understanding by uncovering how specific elements (e.g., strong facilitation, consistent communication, co‐learning, and recruitment strategies) function in practice and intersect with individual motivations, especially as they relate to the field of oncology, to foster engagement. Notably, despite high engagement scores as measured by the REST, participants answered “not applicable” to an average of 20% of the questions. These “not applicable” responses suggest limitations in the REST's fit for all roles and contexts, as well as differences in how participants experience and define engagement [14, 19]. This highlights the importance of complementing quantitative measures with qualitative insights to fully capture the complexity of meaningful involvement.

Both qualitative and quantitative data emphasized the role of patient engagement in shaping the end goal and final product. While PAG members understood the nuances of specific aspects of the OEA project, qualitative analysis suggested a notable disconnect with the ultimate deliverable. This gap led us to select EP7 (involve all partners in the dissemination process) as a focus area for REST assessment. Notably, EP7 was the lowest‐scored engagement principle among PAG members and showed the greatest divergence between PAG and OEA team responses. While PAG members were generally engaged, the greatest opportunity for growth was in the development of the end product and its dissemination. This finding aligns with existing research showing that patient involvement in dissemination and consideration of project deliverables is often limited or tokenistic, emphasizing the need for genuinely collaborative engagement throughout the project lifecycle [30, 31]. Equally important is the use of continuous evaluation to identify divergences between team and PAG member perceptions, as we observed in EP7, enabling targeted improvements and better alignment of engagement strategies.

This mixed‐methods evaluation of PAG engagement identified high levels of perceived engagement, highlighted strong facilitators, and revealed ongoing areas for improvement, despite important limitations. The PAG that was evaluated was focused on a quality improvement initiative at a single institution, and the evaluation occurred at a single time point which may limit the generalizability of our findings to other settings, institutions, or types of advisory groups. However, our sample included a high level of participation (8 out of 10 PAG members in focus groups, full participation in the survey), suggesting that the perspectives captured are broadly representative of this PAG. Thus, any potential selection and representation bias largely stems from the initial processes used to select PAG membership and OEA team employment, rather than from non‐response within our study sample. Moreover, the community‐engagement principle of local relevance over generalizability suggests that evaluation of PAG engagement should occur in the local context [32]. Assessing how engagement is experienced by PAG members within their environment provides valuable insight into what works locally and how engagement practices can be strengthened in similar contexts. Further assessment will be needed to understand how adaptations prompted from our evaluation impacts engagement and how engagement impacted program outcomes and deliverables.

For patient engagement to realize its full potential to improve clinical care and research, patient‐centered strategies such as PAGs must be systematically evaluated to understand how engaged members feel, the factors driving engagement, and alignment of engagement with the initiative goals. Our evaluation identified key successes and challenges in PAG engagement, enabling a focused assessment of perceived engagement and offering actionable insights for future improvement. Importantly, our use of a mixed‐methods approach provided a more comprehensive understanding, capturing not only how engaged members felt, but also the contextual and relational factors that influenced their experience. Future work should compile lessons learned from evaluations such as ours to develop evidence‐based best practices to guide how to meaningfully engage patients and rigorously evaluate engagement.

## Author Contributions

**Lauren Kearney:** conceptualization, methodology, formal analysis, data curation, writing – original draft, visualization. **Tracy Battaglia:** conceptualization, methodology, writing – review and editing, supervision, funding acquisition. **Karina Verma:** formal analysis, writing – review and editing. **Sara Shusterman:** formal analysis, writing – review and editing. **Michelle Hall:** formal analysis, writing – review and editing. **Gemmae Fix:** formal analysis, writing – review and editing. **Katrina Steiling:** formal analysis, writing – review and editing, conceptualization, supervision, funding acquisition.

## Disclosure

The authors alone are responsible for the views expressed in this article and they do not necessarily represent the views decisions, or policies of the Department of Veterans Affairs or the US government.

## Ethics Statement

This study was approved by the Boston University/Boston Medical Center Institutional Review Board.

## Conflicts of Interest

The authors declare no conflicts of interest.

## Supporting information

PAG_Supplement_Updated.

## Data Availability

Given the small sample size and easily identifiable nature of the data, it is not publicly available.
