# Supplementary material for: Evaluating the Process of Patient Engagement: Insights from a Mixed‐Methods Evaluation of a Cancer Center Patient Advisory Group
Source: Health Expect. 2026 Jan 24;29(1):e70581. doi: 10.1111/hex.70581 (PMC12831170; doi:10.1111/hex.70581)
Supplement: Supplementary file 1 — PAG_Supplement_Updated. [file HEX-29-e70581-s001.docx]

Supplement for Evaluating the Process of Patient Engagement: Insights from a Mixed-Methods Evaluation of a Cancer Center Patient Advisory Group

Semi-Structured Guides

Appendix 1a. PAG Evaluation Participant Focus Group Semi-Structured Guide

1. I would like to start by hearing about your experiences serving as a member of the patient advisory group so far.
   1. What had stood out the most about the experience?
   2. What have you liked about the experience?
   3. Was the experience what you thought it would be like?
   4. What do you think could be improved about the experience?
2. We would like to learn more specifically about how this experience has been both so we can improve it for all of you and so we can use a similar approach moving forward in other research studies.
   1. What do you think about the number of meetings and time commitment of the patient advisory group?
      1. Should there be more or less meetings? Should meetings be shorter or longer? Has anyone considered discontinuation because of the burden of the commitment?
   2. Have there been any barriers to you joining the meetings?
      1. Probe for: time of day, costs
   3. What have been the best parts of participating in the patient advisory group? The worst parts?
      1. What times have made you feel most valued? Least valued?
   4. Do you feel that the study team members have taken your input into consideration?
      1. Has the patient advisory group helped make improvements to the research?
   5. Do you feel that you have had control over your involvement in the patient advisory group?
      1. Are you able to voice your thoughts and opinions?
   6. Have there been any downsides to participating in the patient advisory group?

We are interested in hearing how we can improve things over the next year. What changes should we make to how we are conducting meetings with the patient advisory group?

Appendix 1b. OEA Team Member Qualitative Interview

1. Can you please tell us more about your role in the research team? What is your day to day and week to week duties in this research team?
   1. Specifically, what is your role in the patient advisory group?
2. Have you had experience with patient advisory groups or similar community members engaged in research before? How does this compare to your prior experiences?
3. What aspects of the approach to the patient advisory group do you think have been most successful to keep participants engaged?
   1. Probe for: quarterly meetings for feedback on research, monthly meetings or ad hoc meetings to explore cancer processes, email and phone outreach.
4. Where do you think aspects of the patient advisory group have not been successful?
   1. What adaptations have been made to improve the process of the patient advisory group?
5. What has surprised you about the patient advisory group?
   1. What things have gone differently than anticipated or planned?
6. If you were designing this approach again, what changes would you make?

| **Table S1: Results of dimensions of engagement survey** | |
| --- | --- |
| **Question** | **Average (Range)** |
| **I am able to contribute ideas in a productive way** | 3.6 (3-4) |
| **I feel like a valued member of the team** | 3.6 (3-4) |
| **My voice is represented in progress to date** | 3.4 (3-4) |
| **I am able to use my expertise** | 3.2 (2-4) |
| **I am appropriately compensated for my time** | 3.3 (3-4) |
| **The meetings and calls are worth my time** | 3.4 (3-4) |

*1: Strongly Disagree, 2: Disagree, 3: Agree, 4: Strongly Disagree
